# Clinical Case of Feline Leishmaniosis: Therapeutic Approach and Long-Term Follow-Up

**DOI:** 10.3390/vetsci9080400

**Published:** 2022-07-31

**Authors:** Ettore Napoli, Giovanni De Benedetto, Cristina Fazio, Francesco La Russa, Gabriella Gaglio, Emanuele Brianti

**Affiliations:** 1 Department of Veterinary Sciences, University of Messina, 98168 Messina, Italy; enapoli@unime.it (E.N.); gdebenedetto@unime.it (G.D.B.); francesco.larussa@izssicilia.it (F.L.R.); ebrianti@unime.it (E.B.); 2 Independent Researcher, 95100 Catania, Italy; cfazio@gmail.com; 3 Istituto Zooprofilattico Sperimentale della Sicilia “A. Mirri”, 90129 Palermo, Italy

**Keywords:** *Leishmania infantum*, cat, treatment, long term follow-up, allopurinol

## Abstract

**Simple Summary:**

In the present report, clinical, diagnostic, and therapeutical findings observed in a case of feline leishmaniosis (FeL) along with its long-term follow-up data are reported with the aim to provide more evidence-based data on the treatment of this important zoonotic vector-borne disease of cats. The present study represents one of the few reports of FeL with a 28-month follow-up period. This case underlines the effectiveness of allopurinol and highlights how the interruption of treatment frequently leads to relapse, impairing the animal’s health condition and prognosis.

**Abstract:**

The response to allopurinol treatment and survival time of a case of feline leishmaniosis in a FIV co-infected cat is herein reported. In May 2019, a 13-year-old neutered European Shorthair male was referred due to weight loss and exfoliative dermatitis. Lymphadenomegaly and splenomegaly were detected upon clinical inspection, while the presence of several *Leishmania infantum* amastigotes were detected on splenic and lymphonodal fine needle aspiration (FNA). Allopurinol (10 mg/kg PO q 12 h) was administered. After two months, the cat’s clinical symptoms disappeared, and the owners decided to interrupt the therapy. In February, two reddish nodular fleshy neoformations appeared in both eyes, and amastigotes of *Leishmania* were detected by cytology on conjunctival swabs. Allopurinol treatment was re-started at the same rate; the ocular lesions regressed in two weeks, and the owner again decided to interrupt the therapy. In July, the patient had a new relapse, but the owner, tired of continuous relapses, refused further treatment of the disease. The cat’s health condition continued to worsen: in October 2021, the ocular lesions appeared again, and in November the patient died. This case underlines the effectiveness of allopurinol and highlights how interruption of treatment frequently leads to relapse, impairing the animal’s health condition and prognosis.

## 1. Introduction

Leishmaniosis is a vector-borne disease (VBD) caused by protozoan parasites of the genus *Leishmania*, which in Europe was traditionally described in Mediterranean countries and is nowadays regarded as a globally emerging disease spreading to higher latitudes and elevations [1,2,3,4,5,6,7]. Although infected dogs remain the main domestic reservoir host of the disease, an increasing number of other mammalians have been regarded as receptive hosts and potential sources of infection for sand fly vectors [8,9,10]. For instance, in a specific epidemiological scenario, an important role in the establishment of human leishmaniosis outbreaks was played by lagomorphs such as hares or wild rabbits [11,12].

Cats are found to be naturally infected by *Leishmania infantum* [13,14,15,16,17,18], with a prevalence of up to 60.7% in the endemic regions of the Old World [13], which in some cases is higher than for dogs of the same area [19]. Feline leishmaniosis (FeL) normally has a chronic course and may be featured by a plethora of clinical signs, dermatological lesions and lymph node enlargement being amongst the most common [20]. Indeed, it has been estimated that dermatological disorders account for more than half of the clinical manifestations of FeL caused by *L. infantum* [13] and that they may occur in the apparent absence of other obvious signs [18], while on some occasions, dermatological disorders with other systemic signs and pathological alterations have been reported concurrently [20]. The most common non-cutaneous clinical signs include ocular lesions, gingivostomatitis, hepatomegaly, splenomegaly, and decreased appetite [13,21].

There is still no consensus on therapeutic strategies, although several treatments, borrowed from those used in dogs, have been empirically used in cats with a variable degree of effectiveness [22]. In fact, no controlled studies on the efficacy and safety of anti-*Leishmania* drugs have yet been performed on cats. Long-term allopurinol administration as monotherapy is the most common treatment against FeL [16,18,21,22,23,24]. This drug is generally well-tolerated by cats; however, in a few cases, increases of liver enzymes and coprostasis have been observed [24]. The administration of allopurinol once or even twice a day is rather complex in feline patients and makes the owner uncompliant for long-period treatment; relapses after discontinuation or low-dose administration are also common findings [16,22,25,26]. Recently, the use of meglumine antimoniate alone or coupled with allopurinol has been proposed for FeL treatment, appearing to be more effective [25]; however, this approach should cause renal failure in cats with kidney disease [24]. Miltefosine has also been recently adopted as an alternative to antimoniate treatment [24]; nevertheless, it seems that the use of this molecule in cats is potentially associated with transient vomiting episodes, and miltefosine treatment failure in case of co-infections (e.g., FIV, *Toxoplasma gondii*, and *Babesia henselae*) has been reported [18,22].

Most of the FeL cases reported in the literature are limited to the description of clinical signs and pathological alterations, while follow-up data and survival time following treatment have been reported in few cases only [16,22,23,27,28,29,30]. In the present report, clinical, diagnostic, and therapeutical findings observed in a case of FeL along with its long-term follow-up data are reported, with the aim to provide more evidence-based data on the treatment of this important zoonotic vector-borne disease of cats.

## 2. Material and Methods

**Scheme 1:**
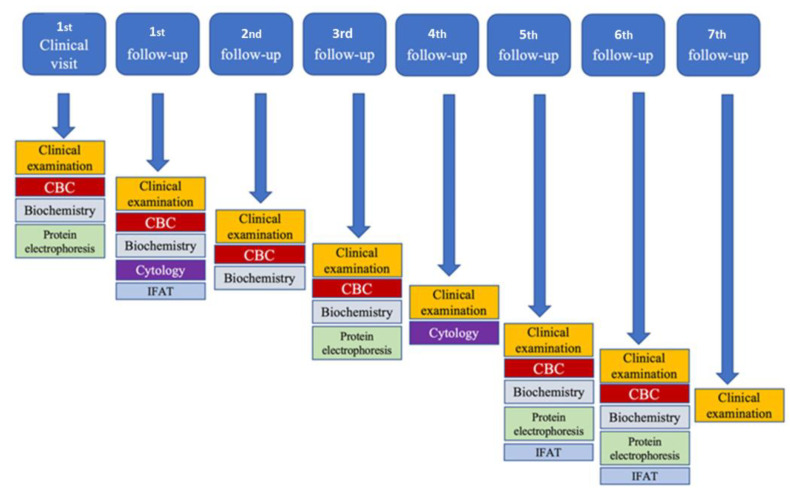
Analyses conducted in each of the follow-up visits according to the animal health condition and treatment response.

The patient was a thirteen-year-old European Shorthair (ESH), neutered male with a 4 kg body weight, living in Catania province (Sicily, southern Italy), an area considered endemic for canine leishmaniosis [31], and it was referred, in May 2019, due to several episodes of vomiting. In the first visit, the patient underwent a standard physical examination and a complete cells blood count using an automated haematology analyser (HeCo Vet C, SEAC, Florence, Italy). Serum proteins (i.e., albumin, globulins), creatinine, and alanine amino-transferase (ALT) were also estimated using an automated UV spectrophotometer (Slim, SEAC, Florence, Italy). Particularly, seven follow-up visits for monitoring the health condition of the animal and treatment response were performed. In the Scheme 1, the analyses conducted in each of the follow-up visits are reported.

Infection by FeLV and/or FIV was tested using an ELISA rapid assay (SNAP Combo FeLV antigen/FIV antibody, IDEXX Laboratories, Westbrook, ME, USA). Smears of the material collected by ultrasound-guided splenic biopsies or fine needle aspiration (FNA) of the popliteal lymph node were stained using May-Grünwald-Giemsa quick stain (Bio-Optica, Milan, Italy) and microscopically observed. An immunofluorescence antibody test (IFAT) for the detection of specific antibodies against *L. infantum* was performed as described by Iatta [32] using a cut-off of 1:80.

## 3. Results

**Figure 1:**
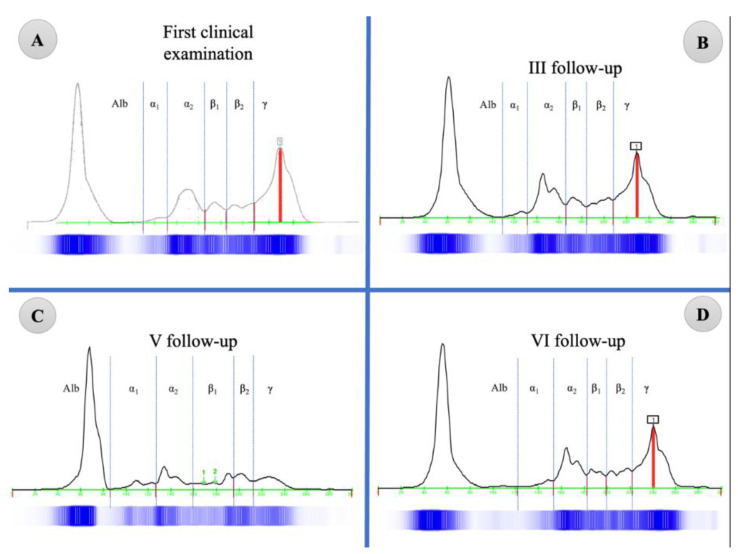
Modifications of the electrophoresis of serum proteins in a cat affected by *Leishmania infantum* and treated with allopurinol 10 mg/kg PO q 12 h. (**A**) At the first diagnosis before treatment with an antibody titre of 1:1280; (**B**) after six months of treatment; (**C**) at nine months follow-up with an antibody titre of 1:640; (**D**) before the final interruption of the therapy with an antibody titre of 1:320.

At the first visit in May 2019, the cat presented thrombocytopenia and slight lymphopenia (Table 1). The biochemistry did not show any alteration of parameters, but urea and creatinine were out of the physiological ranges (Table 2). The electrophoresis showed a significant hypergammaglobulinemia, a slight hypoalbuminemia, and, consequently, a reduced albumin/globulin ratio (Table 3, Figure 1A).

The patient tested positive to the FeLV and FIV ELISA rapid assay, and it was therefore hypothesized that the observed alterations were induced by the FIV infection, and a symptomatic and supportive therapy was prescribed.

**Figure 2:**
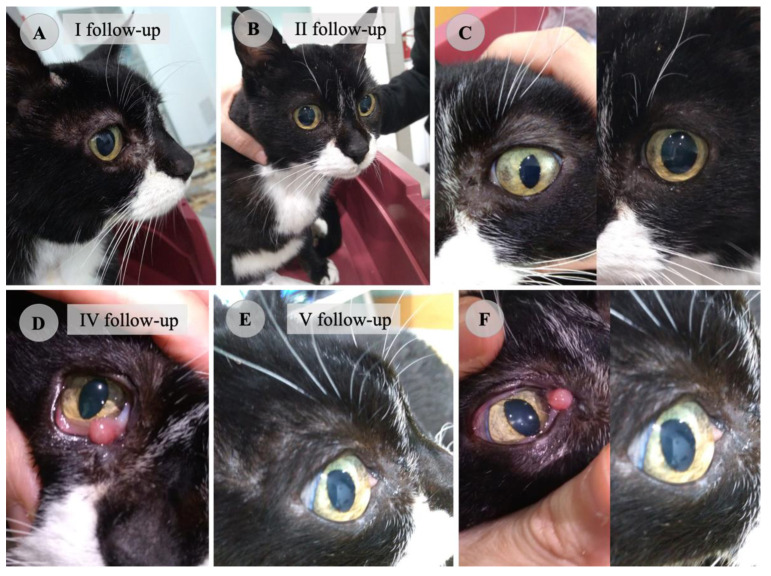
Evolution of clinical signs in a leishmaniotic cat treated with allopurinol 10 mg/kg PO q 12 h. (**A**) First follow-up and (**B**) two-months follow-up. (**C**) Comparison of the same periocular region before and after the treatment. (**D**) First relapse at the IV follow-up, (**E**) the improvement of the lesion after treatment, (**F**) and the resolution of the ocular granuloma after the treatment with allopurinol.

**Figure 3:**
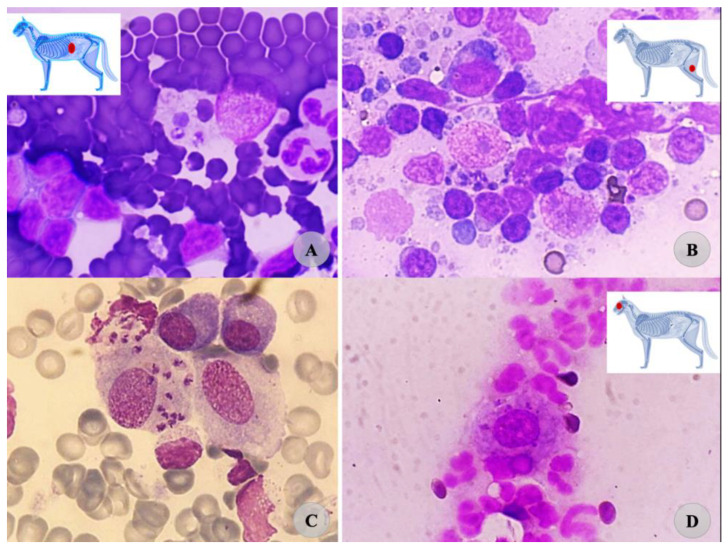
Presence of *Leishmania* amastigotes in macrophages collected through (**A**) FNA of spleen or (**B**–**D**) popliteal lymph node or through (**D**) swabs from ocular lesions.

In November 2019, the cat was referred again due to the worsening of clinical conditions. On that occasion, the cat showed a significant weight loss (i.e., 3.5 kg), sensory depletion, exfoliative dermatitis, and alopecic areas on the eyelids (Figure 2A); moreover, the enlargement of palpable lymph nodes and splenomegaly were observed. Increases of White Blood Cells (WBC) with lymphocytosis, anaemia, and thrombocytopenia were also present (Table 1). Therefore, considering the enlargement of the spleen combined with moderate leucocytosis, ultrasound-guided splenic biopsies and FNA of the popliteal lymph node were performed. The obtained smears showed the presence of several histocytes engulfed by *L. infantum* amastigotes (Figure 2C,D and Figure 3A,B, respectively). The serology for *L. infantum* through IFAT was positive with a titre of 1:1280 (Table 3), and allopurinol (Allopurionolo, Teva, Assago, Italy) at a dose rate of 10 mg/kg PO q 12 h was prescribed.

At the second follow-up, in January 2020, two months after the diagnosis of FeL, the animal gained weight (i.e., from 3.5 kg to 4.5 kg), the lymphadenomegaly, the splenomegaly, and the exfoliative dermatitis disappeared (Figure 2B), and the periocular alopecia significantly decreased (Figure 2C). However, some alterations in CBC and biochemistry were still present (Table 1 and Table 2), and therefore, the allopurinol therapy was continued. In June 2020 (third follow-up), a physical examination was conducted, and the cat did not show any clinical symptoms; however, macrocytosis, hypochromia, leukopenia, and a significant depletion of platelets were still present (Table 1), and the therapy was maintained. The owner, however, was reluctant to continue the per os therapy and, given the absence of evident clinical symptoms, decided to interrupt the allopurinol administration.

In February 2021 (fourth follow-up), a bilateral eye lesion appeared, represented by a reddish nodular, fleshy neoformation; it was observed in the nasal cantus of both eyes (Figure 2D); in the cytological smears of the nodular lesion, several amastigotes within the histocytes cytoplasm were observed (Figure 3D). Therefore, a local therapy with an ophthalmic ointment (i.e., tetracycline 1% and sulfamethazine 5% BID for ten days), combined with the allopurinol therapy, was prescribed. In March (fifth follow-up), a significant regression of ocular lesions was observed (Figure 2E), and the owner decided, against the veterinary prescription, to interrupt the therapy with allopurinol. Serology through IFAT was repeated, and the antibody titre was decreased to 1:640.

In July 2021 (sixth follow-up), the cat had a new relapse with an exasperation of clinical symptoms, showing weight loss (i.e., 3.7 kg), sensory depletion, and lethargy. Furthermore, the CBC and biochemical examination revealed the presence of several alterations (Table 1 and Table 2). The IFAT was repeated, and the antibody titre was 1:320 (Table 3). In October 2021 (seventh follow-up), the ocular lesions appeared again, similarly to those observed in February. The owners, two years after the first diagnosis, tired of the continuous follow-ups, the difficulties connected with the oral administration of allopurinol, and the sudden relapse of the disease after the therapy interruption, refused to continue the diagnostic work and decided to stop animal care. In November 2021, the patient died.

## 4. Discussion

Cats are incorrectly considered to be refractory to *L. infantum* infection, and less attention is therefore given to this parasitic disease compared to dogs. In the past, this perception led to an underestimation of *L. infantum* infection in the cat population. However, in the last decades, more attention has been paid to this VBD in cats, and in fact the number of FeL reports in the literature has progressively increased [13,14,15,16,17,18,33]. Despite the growing interest in FeL, the information available in the literature about the treatment and long-term follow-up of this disease in cats is meagre and limited to few observations [16].

The present study represents one of the few reports of FeL associated with a longer follow-up (i.e., 28-month follow-up period) and a complete panel of clinical and laboratory tests, along with the observation period, clinical signs, pathological findings, disease progression, and effectiveness of treatment, underlining the factual difficulty in the treatment and the fast relapse after the interruption of treatment.

In the present case, FeL was observed in concomitance with an immunosuppressive coinfection due to FIV, as has been frequently observed in other FeL cases [34,35]; in fact, it has been estimated that about half of FeL cases are associated with other comorbidities, FIV and FeLV being the most common [13,34,35]. Co-infections may potentiate disease pathogenesis and alter clinical manifestations, complicating diagnosis and treatment as well as influencing prognosis [36].

Dermatological disorders, which are considered the most common clinical symptoms caused by *L. infantum* in cats [16,20], were also reported in this case. However, the skin disorders herein observed are not so common; in fact, nodular lesions are regarded as the coarsest skin disorder in FeL, while alopecic and exfoliative dermatitis are less reported [20]. The dermatological lesions reported herein were localized on the eyelid and periocular region, unlike other studies where the same lesions in different areas of the head were reported [16,18], the distal part of the body [18,37], the thorax and abdomen areas [38], and on bony prominences [21].

It is worth noting that we describe two nodular fleshy neoformations of conjunctivas that appeared for the first time in February 2021 and quicky disappeared after just two weeks of therapy; the same lesions reappeared in October soon after the interruption of treatment. This clinical sign is really rare and has only been observed in a few occasions [38]; in fact, among ocular lesions in the literature, uveitis [18,23,24,39], chorioretinitis [18], corneal opacification [23], glaucoma [26], blepharitis [16], and chemosis [18] were mainly observed. Whereas generalized or focal lymphadenopathy appears to be a common finding [16,18,21,33,40], other clinical manifestations such as footpad hyperkeratosis [18], hepatomegaly [26], and splenomegaly [39,40] are rarely found and may represent a further diagnostic challenge to veterinarians; in fact, as reported herein, the presence of splenomegaly was combined with the localized lymphadenomegaly (i.e., popliteal lymph node). In fact, in the present case, the FeL infection was taken into account just after the accidental detection of amastigotes in the spleen and lymph node FNA.

It is worth nothing that, as already observed for CanL [41,42], in the course of FeL, clinicopathological alterations such as anaemia, leucocytosis, hyperglobulinemia, and hypoalbuminemia [16] and chronic renal failure are common findings. However, some authors consider the presence of leucocytosis [18] or leukopenia [22,43] to be inconsistent findings with FeL [24]. In the present report, any significant laboratory abnormalities were observed in the CBC, except for an increase of WBC in November and in January, which were not necessarily related to the *L. infantum* infection. Azotaemia and creatinine were considerably increased in all the samplings; moreover, the value of these two parameters constantly increased during the observation period. In the literature, the increase of azotaemia [18,22,26] has been reported in association with FeL and renal failure, even if in the present case there is no evidence of renal disease.

In cats, hyperproteinaemia and hypergammaglobulinemia should be suggestive of several infectious diseases, such as FIV, FeLV, and feline infectious peritonitis (FIP), as well as FeL [16,18,21,22,35,44]. It is a fact that, in the case of cats with hyperproteinaemia and hypergammaglobulinemia, clinicians did not include FeL in the differential diagnosis, as reported herein.

The antibody titre observed in this patient was significantly higher compared to other similar studies [16]. It is worth noting that this higher titre is not related to the disease clinical presentation in the patient and that even if the cats underwent several relapses during the observation period, the antibody titres decreased progressively from 1:1280 to 1:320. This finding is not surprising; in fact, high antibody titres in cats are not always related to the presence of clinical signs [45].

In the present case, the long-term allopurinol administration in monotherapy was safe and effective, resulting in the resolution of the lesions; moreover, the resolution of the clinical symptoms was faster compared to those observed in a similar case in which the complete healing of the clinical lesions appeared in about seven months [16]. Although allopurinol is generally well tolerated and side effects in cases were only observed on a few occasions [16,39,43,44,45,46], the administration of an oral therapy in cats is normally not well accepted by the owner and the patient. Indeed, the difficulty in administering the drug per os, especially for a long period in cats, generally leads to the interruption of the therapy. However, as observed in the case herein reported, the allopurinol administration guarantees a long survival period, as already observed [16]; this finding is in contrast with a study in which the estimated median survival time is three months after the first diagnosis and the life expectancy of *L. Infantum*-infected cats is not significantly influenced by therapy [47].

Nevertheless, as expected, the treatment was not effective in eliminating the parasite, as demonstrated by cytological testing in the subsequent follow-ups. Moreover, the sudden relapse at the interruption of the therapy could support the parasitostatic effect of allopurinol and the sudden reactivation of the previous infection when the drug is not administered. The reappearance of lesions in the same anatomical area just after the interruption of the therapy suggests that the relapse is due to the reactivation of the previous infections, especially those that occurred in the months of non-vectorial transmission. However, during these two years of observation, the owners did not administer any preventative measure against sand flies, and new infections cannot be ruled out.

Treatment of FeL should always be attempted, since it provides an amelioration of health conditions and therefore animal welfare. Additionally, treatment does provide a reduction of the parasite load, which, in turn, may result in a lower infectivity to sand fly vectors. Nevertheless, it is also essential to adopt preventative strategies (e.g., pyrethroids with an anti-feeding effect on sand flies) in infected/diseased cats, as these protect the treated animal from new infections and, importantly, block the reservoir role of infected animals to sand files [14,24].

## 5. Conclusions

Even if *Leishmania* spp. is acknowledged today as being a possible cause of disease in cats, it is usually still not considered in the diagnostic routine of veterinarians.

Despite scepticism about the effect of the FeL therapy on the health status and survival time still existing, it is important to highlight that a specific therapy guarantees a better quality of life and a longer survival time. In addition, treatment significantly reduces parasite load, which, in turn, results in a better control of transmission, since infected cats may serve as a source of infection for sand fly vectors. Veterinarians must be aware of the susceptibility of cats to *Leishmania* infection, the different clinical presentations of the disease in cats compared to dogs, and the importance of treating infected patients and of proposing, like for dogs, preventative strategies by means of repellent products.

## Figures and Tables

**Table 1:** Cell blood count analyses, including red blood cells (RBC), haemoglobin (HGB), haematocrit (HCT), Mean Corpuscular Volume (MCV), Mean Corpuscular Hemoglobin (MCH), Mean Corpuscolar Hemoglobin Concentration (MCHC), white blood cells (WBC), and platelets (PLT) performed in a cat affected by *Leishmania infantum* and treated with allopurinol 10 mg/kg PO q 12 h at different time points; ^+^/^−^, increasing and decreasing values.

Parameter	Reference Range	Unit	First Visit	I Follow-Up	II Follow-Up	III Follow-Up	V Follow-Up	Vi Follow-Up
**WBC**	6.00–19.00	10^3^/uL	6.53	27.1 ^+^	72.6 ^+^	4.91	4.49	4.90
Neutrophils	2.50–12.00	10^3^/uL	4.21	---	---	2.40	2.50	2.60
Lymphocytes	2.20–8.00	10^3^/uL	2.11	12.57	64.3	2.31	2.1	3.1
Monocytes	0.20–1.00	10^3^/uL	0.15	---	---	0.12	0.20	0.10
Eosinophils	0.20–1.50	10^3^/uL	0.05	---	---	0.08	0.20	0.0
Basophils	0.00–0.10	10^3^/uL	0.01	---	---	0.00	0.9	0.0
**RBC**	5.00–10.10	10^6^/uL	7.14	5.43	5.18	6.97	7.78	7.64
HGB	8.0–15.0	g/dL	11.3	8.8	12.4	10.3	11.0	10.7
HTC	25.0–45.0	%	39.1	34.7	41.8	35.6	36	37.4
MCV	41.0–45.0	fL	54.8 ^+^	60.0 ^+^	67.7 ^+^	51.1 ^+^	46 ^+^	49.0 ^+^
MCH	13.0–17.0	Pg	15.8	13.05	20	14.8	14	14.0
MCHC	31.0–36.0	g/dL	28.9	24.4	29.6	28.9	30.3	28.6
**PLT**	150–500	10^6^/uL	23 ^−^	100 ^−^	198	84 ^−^	146 ^−^	97 ^−^

**Table 2:** Biochemistry analyses of the leishmaniotic cat during the study period. (AST = aspartate aminotransferase; ALT = alanine amino-transferase; A/G = albumin and globulin ratio); ^+^/*, increasing and decreasing values.

Parameter	Reference Range	Unit	First Visit	I Follow-Up	II Follow-Up	III Follow-Up	V Follow-Up	VI Follow-Up
Azotemia	10.0–30.0	mg/dL	62 ^+^	41 ^+^	84 ^+^	80 ^+^	12 *	66 ^+^
Creatinine	<1.5	mg/dL	2.2 ^+^	2.3 ^+^	3.8 ^+^	3.3 ^+^	0.7 *	3.5 ^+^
Urea/Creatinine			28.2	17.8	22.1	---	17.1	18.9
Total cholesterol	90.0–200.0	mg/dL	109	---	143.6	164	228 *	148
AST	<80	UI/L	30	---		32	45	32
ALT	<80	UI/L	47	---	94	80	140	65
Total bilirubin	<0.6	mg/dL	0.48	---	0.42	0.22	0.58	0.20
Alkaline phosphatase	<200	UI/L	33	---	35	57	4479 *	87
Total protein	5.3–7.9	g/dL	6.7	---	7.87	6.7	6.6	6.3
Globulin		mg/dL	4.1		4.66	3.8	3.5	3.5
Albumin	2.1–3.4	g/dL	2.6	---	3.21	2.9	3.1	2.8
A/G			0.6		0.7	0.8	0.9	0.8

**Table 3:** Values of serum protein electrophoresis performed in a cat affected by FeL during the study period.

Parameter	Reference Range	Unit	First Visit	I Follow-Up	II Follow-Up	III Follow-Up	V Follow-Up	VI Follow-Up
Albumin	44.0–56.0	%	39.9	---	---	43.2	55.6	44.4
Alpha 1	2.7-5.0	%	1.5	---	---	1.90	5.8	2.4
Alpha 2	6.3–12.5	%	13.8	---	---	16.50	12.0	14.2
Beta 1	2.0–8.0	%	5.9	---	---	5.70	9.3	5.6
Beta 2	7.0–11.5	%	7.6	---	---	7.50	6.9	8.0
Gamma	12.0–22.00	%	32.3	---	---	26.60	10.4	25.4
Albumin	2.1–3.4	g/dL	2.6	---	---	2.90	3.1	2.8
Alpha 1	0.1–0.6	g/dL	0.1	---	---	0.10	0.3	0.2
Alpha 2	0.3–1.3	g/dL	0.9	---	---	1.00	0.7	0.9
Beta 1	0.3–0.7	g/dL	0.4	---	---	0.40	0.6	0.4
Beta 2	0.2–1.3	g/dL	0.5	---	---	0.50	0.4	0.5
Gamma	0.8–1.5	g/dL	2.2	---	---	1.80	0.5	1.6
Total protein		g/dL	6.68	---	---	6.70	5.58	6.28
Alb/Glob rate	>0.9		0.64	---	---	0.76	1.25	0.86
**IFAT Leishmania**			---	1:1280	---	1:1280	1:640	1:320

## Data Availability

All the data are available from the corresponding author on reasonable request.
